# Antimicrobial resistance in wildlife: detection of antimicrobial resistance genes in Apennine wolves (*Canis lupus italicus* Altobello, 1921) from Central Italy

**DOI:** 10.1007/s11259-024-10354-8

**Published:** 2024-03-19

**Authors:** A. Di Francesco, D. Salvatore, A. Ranucci, M. Gobbi, B. Morandi

**Affiliations:** 1 Department of Veterinary Medical Sciences (DIMEVET), University of Bologna, Ozzano dell’ Emilia (BO), Bologna, Italy; 2 Istituto Zooprofilattico dell’Umbria e delle Marche ‘Togo Rosati’ Perugia, Perugia, Italy

**Keywords:** Antimicrobial resistance, Antimicrobial resistance genes, Wolf, *Canis lupus italicus*, PCR, Italy

## Abstract

The aim of this study was to molecularly investigate the presence of antimicrobial resistance genes (ARGs) in organ samples from 11 Apennine wolves (*Canis lupus italicus*) collected in Central Italy. Samples from lung, liver, spleen, kidney, tongue and intestine were investigated by PCRs targeting the following genes: *tet*(A), *tet*(B), *tet*(C), *tet*(D), *tet*(E), *tet*(G), *tet*(K), *tet*(L), *tet*(M), *tet*(O), *tetA*(P), *tet*(Q), *tet*(S), *tet*(X), *sul1*, *sul2*, *sul3*, *bla*_CTX−M_, *bla*_SHV_, *bla*_TEM_ and *mcr*-1. A PCR positivity was highlighted for 13 out of the 21 tested genes; no positive results were obtained for *tet*(C), *tet*(D), *tet*(E), *tet*(G), *sul3*, *bla*_CTX_, *bla*_SHV_ and *mcr*-1 genes. All 11 animals sampled showed positivity for one or more resistance genes. The results confirm the potential role of the wolf as an indicator and/or vector of antimicrobial-resistant bacteria or ARGs.

## Introduction

Antimicrobial resistance (AMR) seriously threatens human health worldwide, impairing our capacity to treat an increasing number of infections, with economic and social consequences (Vittecoq et al. 2016). This complex phenomenon recognises multiple actors, involving humans, animals and the environment, including wildlife that could act as an epidemiological link between the different compartments (Plaza-Rodriguez et al. 2021). Although wild animals are unlikely to be treated with antibiotics, compared to domestic animals or humans, antimicrobial-resistant bacteria and/or antimicrobial resistance genes (ARGs) have been detected in numerous wildlife species (Radhouani et al. 2014; Ramey 2021). Multi-drug-resistant strains were more frequently found in wild animals that used anthropised sites, as a consequence of anthropic AMR pollution of the ecosystems they inhabited (Laborda et al. 2022). Waste from anthropogenic sources potentially containing antibiotics, antimicrobial-resistant bacteria or ARGs, such as hospitals, wastewater treatment plants, pharmaceutical industries and/or animal husbandry facilities, can contaminate the natural environmental compartment, of which wildlife is an integral part (Baros Jorquera et al. 2021). Once antimicrobial-resistant bacteria or ARGs have been acquired by wildlife hosts, they can be transmitted to other environments, humans included, contributing to AMR spread locally and worldwide (Laborda et al. 2022).

Among wild animals, those with high ecological plasticity, such as the ability to survive in numerous different contexts, the wide territorial dispersion and the predatory attitude, might be indicators and/or vectors of AMR environmental contamination. In this regard, recent studies showed the presence of antimicrobial-resistant bacteria or ARGs in Iberian (*Canis lupus signatus*) and Apennine (*Canis lupus italicus*) wolves (Simões et al. 2012; Gonçalves et al. 2012; Smoglica et al. 2020, 2023a) as well as in golden jackals in Italy (Di Francesco et al. 2023b).

The molecular detection of target ARGs from biological or environmental samples was recently proposed as an alternative to culture-dependent methods (Smoglica et al. 2020; Galhano et al. 2021). The molecular approach presents a series of favourable aspects such as high speed, independence from the vitality of the microorganisms and the possibility of bypassing the difficulties of isolation from carcasses in an advanced state of decomposition, a frequent condition in wildlife (Di Francesco et al. 2020; Francesco et al. 2023a). However, it does not allow determining the bacterial sources of the ARGs detected, as well as tracing the evolutionary changes in the “possessors” of resistance genes.

The aim of this study was to examine, by PCR, organ samples from 11 Apennine wolves for the presence of ARGs against antimicrobials extensively used both in human and veterinary fields.

## Materials and methods

From January 1 to June 30, 2023, 11 carcasses of Apennine wolves (6 females and 5 males, average age 3 years), involved in road accidents in the Marche and Umbria regions (Central Italy), were submitted to the Istituto Zooprofilattico Sperimentale Umbria and Marche ‘Togo Rosati’ to determine the cause of death. During necropsy, samples from the lung, liver, spleen, kidney, tongue (only in 8 animals) and intestine were collected, stored at − 20 °C and sent to the Department of Veterinary Medical Sciences (Ozzano dell’Emilia, Bologna, Italy) for molecular investigations. Only intact organs were taken into consideration to exclude contamination.

Total DNA was extracted from each organ sample using the DNeasy Tissue Kits (Qiagen, Hilden, Germany) following the supplier’s recommendations. One extraction control, consisting of kit reagents only, was included.

The DNA samples were investigated for the presence of ARGs against antimicrobials extensively used both in human and veterinary fields, such as tetracyclines, sulphonamides, β-lactams and colistin. Specifically, the following genes were tested: *tet*(A), *tet*(B), *tet*(C), *tet*(D), *tet*(E), *tet*(G), *tet*(K), *tet*(L), *tet*(M), *tet*(O), *tet*(S), *tetA*(P), *tet*(Q), *tet*(X), *sul1*, *sul2*, *sul3*, *bla*_CTX−M_, *bla*_SHV_, *bla*_TEM_ and *mcr*-1. Each gene was amplified by an individual PCR, using specific primers (Di Francesco et al. 2023). The following PCR protocols were carried out: 5 min of initial denaturation at 94 °C, followed by 35 cycles at 94 °C for 1 min, 50 °C [*tet*(K)], 51 °C [*tetA*(P), *tet*(S) and *sul3*], 53 °C [*tet*(B), *tet*(D), *tet*(E), *tet*(M), *tet*(Q), *tet*(X), *bla*_SHV_ and *mcr*-1], 55 °C [*tet*(A), *tet*(C), *tet*(G), *tet*(L), *tet*(O), *sul2*, *bla*_CTX−M_ and *bla*_TEM_] or 59 °C (*sul1*) for 1 min, and 72 °C for 1 min. A final extension step of 10 min at 72 °C completed the reaction. A specific positive control was used for each ARG tested, using DNAs extracted from *Escherichia coli* field strains, containing antimicrobial resistance plasmids. The extraction control and a distilled water negative control were also included. The PCR products were analysed by 2% agarose gel electrophoresis; the DNA bands were stained with Midori Green Advance (Nippon Genetics Europe GmbH, Düren, Germany) and then visualised using ultraviolet (UV) trans-illumination. All amplicons were purified using the High Pure PCR Product Purification Kit (Roche, Mannheim, Germany), and both DNA strands were sequenced by Sanger method (Bio-Fab Research, Rome, Italy). The sequences obtained were compared with the public sequences available using the BLAST server in the GenBank database (National Center for Biotechnology Information 2023, https://www.ncbi.nlm.nih.gov/).

## Results

The results are shown in Table 1.

**Table 1:** Antimicrobial resistance genes detected in the samples tested

Animals	Lung	Liver	Spleen	Kidney	Tongue	Intestine
CL23-02	*tet*(L)	*tet*(L), *tet*(M)	*tet*(K), *tet*(L), *tet*(M)	−	n.a.	*tet*(K), *tet*(L), *tet*(M), *tet*(O)
CL23-07	*tet*(L), *tetA*(P)	*tet(*A), *tet*(L), *tetA*(P)	*tet*(L), *tet*(O), *tetA*(P)	*tet*(L), *tetA*(P)	n.a.	*tet*(M)
CL23-08	*tet*(K), *tet*(M), *tet*(O), *sul2*	−	*tet*(M), *sul2*	*tet*(L), *tet*(M)	n.a.	*tet*(A), *tet*(B), *tet*(M), *tet*(O), *tetA*(P), *sul1*, *sul2*, *bla*_TEM−1_
CL23-09	*tet*(A), *sul1*, *sul2*	*tet*(A), *tet*(L), *tet*(M), *tet(*O), *tetA*(P), *sul1*, *sul2*, *bla*_TEM−1_	*tet*(A), *tet*(L), *tet*(M), *tet*(O), *tetA*(P), *tet*(S), *sul2*	*tet*(A), *tet*(L), *tet*(M), *tet*(O), *tet*(S)	*tet*(A), *tet*(B), *tet*(K), *tet*(L), *tet(*M), *tet*(O), *tetA*(P), *tet*(X), *sul1*, *sul2*, *bla*_TEM−1_	*tet*(A), *tet*(L), *tet*(M), *tet*(O), *tetA*(P), *tet*(S), *sul1*, *sul2*, *bla*_TEM−1_
CL23-10	*tet*(A)	*tet*(A)	*tet*(L), *tetA*(P), *tet*(X)	*tet*(A), *tet*(M), *tetA*(P)	*tet*(A), *tet*(B), *tet*(K), *tet*(L), *tet*(M), *tet*O), *tetA*(P), *tet*(X)	*tet*(A), *tet*(M), *tet*(O), *tetA*(P), *sul2*
CL23-11	−	−	−	−	*tet*(K), *tet*(L), *tet*(M), *tet*(O), *tet*(Q), *tet*(S)	*tet*(L), *tet*(M), *tetA*(P)
CL23-12	*tet*(A), *tet*(B), *tet*(L), *tet*(M), *tet*(O), *tetA*(P), *tet*(Q), *tet*(X), *sul2*	*tet*(K), *tet*(M), *tetA*(P)	*tet*(B), *tet*(M), *tet*(AP)	*tet*(M)	*tet*(A), *tet*(L), *tet*(M), *tet*(O), *tetA*(P), *tet*(S), *sul2*	*tet*(A), *tet*(B), *tet*(L), *tet*(M), *tetA*(P)
CL23-13	*tet*(L), *tet*(M), *sul2*	*tet*(M)	*tet*(K)	*tet*(M)	*tet(*L), *tet*(M), *tetA*(P), *tet*(S), *sul2*	−
CL23-14	*tet*(K), *sul2*	−	−	−	*tet*(A), *tet*(B), *tet*(K), *tet*(M), *tet*(S), *sul1*, *sul2*	*tet*(A), *tet*(B), *tet*(L), *tet*(M), *tetA*(P), *tet*(S), *sul1*, *sul2*
CL23-15	−	−	*tet*(X)	**−**	*tet*(A), *tet*(M), *tet*(O), *tetA*(P), *tet*(Q), *tet*(X)	*tet*(A), *tet*(M), *tetA*(P), *tet*(Q), *tet*(X), *sul1*, *sul2*
CL23-16	*tet*(M), *tet*(S), *tet*(X)	−	*tet(*K)	*tet*(M), *tetA*(P), *tet*(S), *tet*(X), *sul1*	*tet*(A), *tet*(B), *tet*(K), *tet*(L), *tet*(M), *tet*(O), *tetA*(P), *tet*(S), *sul2*	*tet*(A), *tet*(B), *tet*(K), *tet*(L), *tet*(M), *tet*(O), *tetA*(P), *tet*(S), *tet*(X), *sul2*

n.a.: not available

A PCR positivity was highlighted for 13 out of the 21 tested genes. In particular, *tet*(A), *tet*(B), *tet*(K), *tet*(L), *tet*(M), *tet*(O), *tetA*(P), *tet*(Q), *tet*(S), *tet*(X), *sul1*, *sul2* and *bla*_TEM_ amplicons were observed, whereas no positive results were obtained for *tet*(C), *tet*(D), *tet*(E), *tet*(G), *sul3*, *bla*_CTX_, *bla*_SHV_ and *mcr*-1 genes. Amplicon identity was confirmed by comparison between the sequences obtained and the corresponding sequences available in the GenBank database, showing 99–100% nucleotide similarity. Regarding the *bla*_TEM_ amplicon, sequence analysis did not highlight the amino acid residues that are most frequently involved in conferring the extended-spectrum β-lactamase (ESBL) phenotype to TEM-type enzymes (Bradford 2001). The comparison with the corresponding sequences available in the GenBank database allowed the amplicon to be identified as *bla*_TEM−1_.

Since the sequences obtained were identical to each other for each of the 13 amplified ARGs, a representative sequence for each gene was deposited in the GenBank database under accession numbers PP096854-PP096863 and PP032883-PP032885.

All 11 animals sampled showed positivity for one or more resistance genes. In particular, 100% of the animals were positive for at least one *tet* gene, 73% for at least one *sul* gene and 18% for *bla*_TEM−1_ gene. With respect to the gene frequencies, *tet*(M) was found in 100%, *tet*(L) and *tetA*(P) in 91%, *tet*(K) and *tet*(O) in 82%, *tet*(A) and *sul2* in 73%, *tet*(B) and *tet*(S) in 54.5%, *tet*(X) and *sul1* in 45%, *tet*(Q) in 27% and *bla*_TEM−1_ in 18% of the 11 animals tested.

Considering the sampled organs, the positivity rate was 100% for the tongue, 91% for the intestine, 82% for the lung and spleen, 64% for the kidney and 54.5% for the liver.

## Discussion

In Italy, the wolf was present over much of the territory until approximately two centuries ago. In the 19th and 20th centuries, it suffered a drastic decline due to human persecution, deforestation and the decrease of wild ungulates, which led to its disappearance from the Alps, most of the peninsula and Sicily. The historical minimum dates back to the 1970s, when the wolf’s range of presence was represented by small nuclei separated from each other, with a total of approximately 100 subjects, distributed along the central-southern Apennine ridge (Zimen and Boitani 1975).

In the last two decades, the status of the wolf in Italy, as well as in other European countries (Chapron et al. 2014), has changed greatly. From a condition of extreme precariousness and fragmentation of the population, there has been a constant increase in size and distribution area, which has led the wolf to permanently colonise even areas characterised by significant anthropogenic activity. This phenomenon could be explained by a series of social and ecological factors, such as international conventions (Bern Convention 1979, http://www.coe.int/en/web/bern-convention; Eu Habitats Directive 92/43/EEC, https://eur-lex.europa.eu) that, implemented at national level, introduced a fully protected status in Italy (Legge 5 agosto 1981 n. 503; Legge nazionale 11 febbraio 1992 n. 157; D.P.R. 8 settembre 1997 n. 357; https://www.normattiva.it), as well as the progressive abandonment of agricultural land, which has been recolonised by large carnivores and their wild ungulate prey (Cimatti et al. 2021).

Between 2020 and 2021, the Italian Institute for Environmental Protection and Research (ISPRA) coordinated the first national wolf monitoring, which allowed the number of individuals and their distribution to be estimated (https://www.isprambiente.gov.it/it/attivita/biodiversita/monitoraggio-nazionale-del-lupo/file-monitoraggio/report-nazionale-lupo-20_21.pdf). Overall, the presence of approximately 3,300 wolves was estimated in Italy, with 950 moving in the Alpine regions and almost 2400 being distributed along the remaining part of the peninsula. These data show that the wolf has reached practically every real possibility of spreading in peninsular Italy, permanently also occupying territories different from those traditionally inhabited, such as anthropised plain environments.

In the same way as for other wild carnivores (Brown et al. 2023; Garcês and Pires 2023), anthropogenic pressure could be the key factor to explain the results of recent studies reporting antimicrobial-resistant bacteria and/or ARGs in wolves. In this regard, Simões et al. (2012) and Gonçalves et al. (2012) described *Escherichia coli* isolates from faecal samples of Iberian wolves as showing a phenotype of multiresistance, including resistance to tetracycline, ampicillin, streptomycin and β-lactams. In Italy, the occurrence of the tetracycline resistance gene *tetA*(P) was reported for faecal samples from Apennine wolves (Smoglica et al. 2020). In another recent study, 12 antimicrobial-resistant bacteria were isolated from tissue samples and exudates from 4 Apennine wolves, 6 bacteria of which showed multi-drug resistance to critically important antibiotics (Smoglica et al. 2023a).

In this study the presence of ARGs against antimicrobials extensively used both in human and veterinary fields was investigated.

All animals tested showed positivity for one or more resistance genes (Table 1).

The highest positivity was observed for *tet* genes (100% of the animals tested), which is in line with the widespread tetracycline resistance phenomenon (Wang et al. 2017) due the broad-spectrum activity, low cost and low toxicity. This has led to the intensive use of tetracycline in human and animal infection therapy, including as growth promoters in food animal production systems until 2006, when this practice was stopped in the EU [Regulation (EC) No 1831/2003 of the European Parliament and of the Council of 22 September 2003 on Additives for Use in Animal Nutrition (Text with EEA Relevance). http://data.europa.eu/eli/reg/2003/1831/oj]. The high frequency of the *tet*(M) gene, detected in 100% of the animals tested, is not surprising since this gene has been detected in at least 81 different bacterial genera (https://faculty.washington.edu/marilynr/tetweb2.pdf; https://faculty.washington.edu/marilynr/tetweb3.pdf), probably because of its association with conjugative transposons that appear to have a lower host specificity than plasmids (Roberts 2005).

The sulfonamides were the first synthetic compounds deployed as antibacterial drugs. The broad-spectrum bacteriostatic activity of these chemicals against both Gram-positive and Gram-negative bacteria led to their use in both human and veterinary medicine worldwide (Ovung et al. 2021), resulting in a high prevalence rates of sulphonamide resistance observed in mainly Gram-negative bacteria isolated from animals and humans (Pavelquesi et al. 2021). In this study, *su1* and *sul2* genes were detected in 45% and 73% of the 11 animals tested, respectively. No positive results were obtained for *sul3* gene, according to the literature reporting a higher frequency of *sul1* and *sul2* genes (Wang et al. 2014).

β-lactam antibiotics are among the most commonly prescribed antibiotics due to their minimal side effects and broad antibacterial spectrum. Resistance to the β-lactams continues to increase, especially in Gram-negative organisms, posing a public health concern exacerbated by the rapid evolution of extended-spectrum β-lactamases (ESBLs) which are a group of enzymes that confer resistance to most β-lactam antibiotics, including expanded-spectrum cephalosporins and monobactams (Castanheira et al. 2021). In this study, 18% of the animals tested showed *bla*_TEM−1_ gene encoding the TEM-1 β-lactamase. This gene needs only a few specific single nucleotide polymorphisms to evolve into a gene encoding an ESBL (Muhammad et al. 2014).

Colistin is an antimicrobial agent of the polymyxin class. Colistin resistance is a globalized public health concern because it has been considered a drug of the last-line resort to treat multidrug-resistant deadly infections, in particular by strains resistant to carbapenems. In this study *mcr*-1, the most commonly encountered mobil colistin resistance gene, was tested, without obtaining positive results.

The high percentage of positivity observed for the tongue (100%) and intestine (91%) could be attributed to frequent findings of ARGs associated with commensal gut microbiota (Arnold et al. 2016). In this regard, the gut microbiome has been studied as a reservoir of ARGs that can be transferred to bacterial pathogens via horizontal gene transfer of conjugative plasmids and mobile genetic elements, and therefore confer antimicrobial resistance to virulent and clinically relevant strains (Crits-Christoph et al. 2022).

Interestingly, ARGs were detected in some lung, liver, spleen and kidney samples. Cross-contaminations could be excluded because only intact organs were examined, and DNA extraction was performed on internal fragments of each organ that had been removed with a disposable scalpel blade. Confirming this, some genes were detected only in the tongue and/or intestine [e.g., CL23-11], and different genes were highlighted in the intestine and in the other organs tested, respectively [e.g., CL23-07]. However, the finding of only resistance genes does not allow their certain association with bacterial infections.

The results of the present study confirm that the wolf could contribute to AMR spread. The available data do not allow us to state whether the wolves examined in this study came from anthropised areas or from sites close to animal husbandry facilities. However, considering the wolf’s wide territorial dispersion and the involvement of the wolves tested in road accidents, we cannot exclude this hypothesis. Another factor to take into consideration as a source of AMR is the wolf’s predatory activity aimed at wild and domestic prey. Studies on wolf nutrition in the northern Apennines have highlighted that the quantitative ratios of domestic and wild ungulates in the diet are generally inversely correlated; a low consumption of domestic ungulates was detected in areas with high densities of wild ungulates, such as wild boar, roe deer, red deer and chamois (Meriggi and Lovari 1996; Meriggi et al. 2011). Regarding the predation of livestock, Ispra estimated the impact of the wolf on livestock activities in Italy in the period 2015–2019, reporting 17,989 predation events for a total of 43,714 livestock (https://www.isprambiente.gov.it/public_files/StimaImpattoLupoAattivitaZootecniche.pdf). Among the preyed animals, 82.0% were sheep and goats, 14.2% were cattle, 3.2% were equines, 0.1% referred to pigs, 0.1% concerned avian species, and 0.4% were represented by predation on other species or were undetermined cases. The predatory activity, as a possible source of AMR for the wolf, is anyway related to human activities, directly in livestock due to the administration of antimicrobials (Woolhouse et al. 2015) or indirectly in wild prey due to environmental contamination through wastewater or manure (Martinez 2009) and/or the interface with livestock (Smoglica et al. 2023b).

In conclusion, the social and ecological factors that have led to a considerable increase in the number of wolves in Italy and the expansion of its range expose the wolf to direct or indirect anthropogenic pressures, resulting in its possible role as an indicator and/or vector of antimicrobial-resistant bacteria or ARGs. People who may have professional or other contact with wolves should consider the risk of transmission of zoonotic antibiotic-resistant agents or transmissible resistance determinants and take appropriate preventive measures.

Considering the small number of studies on this topic, further investigations on a larger sample of wolves are needed.

Furthermore, the detection of ARGs in biological samples by molecular methods, although it does not allow us to trace the bacterial species involved, seems to be an effective tool in studying the dynamics of AMR diffusion in the environmental compartment, of which wildlife is a part.

## Data Availability

No datasets were generated or analysed during the current study.
